# Preoperative Respiratory Training Before Cardiac Surgery: Feasibility and Longitudinal Pulmonary Function Changes in Elderly Patients

**DOI:** 10.1002/pri.70286

**Published:** 2026-07-16

**Authors:** Andranik Petrosyan, Emilie Besson, Nathalie Grand, Julien Lanoiselee, Khalil Raissouni, Kasra Azarnoush

**Affiliations:** ^1^ Department of Cardiac Surgery University Hospital of Saint‐Étienne Saint‐Étienne France; ^2^ Department of Anesthesia University Hospital of Saint‐Étienne Saint‐Étienne France

**Keywords:** atelectasis prevention, FEV1, forced vital capacity, incentive spirometry, preoperative respiratory preparation, pulmonary function improvement

## Abstract

**Objective:**

The objective of this study was to evaluate the feasibility and short‐term spirometric changes observed during a 3‐week physiotherapist‐supervised incentive‐spirometry program in elderly patients awaiting elective cardiac surgery.

**Methods:**

This prospective, single‐center observational study included patients aged ≥ 65 years scheduled for elective cardiac surgery. Patients completed a 3‐week home‐based respiratory training program using an incentive spirometer (TriFlo 2), comprising six daily sessions and supervised twice weekly by physiotherapists. Pulmonary function, including forced vital capacity (FVC), forced expiratory volume in 1 s (FEV1), and the Tiffeneau index, was evaluated at the baseline, after the training program, and 30 days after surgery.

**Results:**

A total of 27 patients completed the preoperative training phase with high adherence, and 21 patients had complete three‐phase longitudinal spirometric data. No adverse events related to respiratory training were reported. FVC increased significantly after the training program (4.22 ± 1.16 L to 5.09 ± 1.96 L; *p* = 0.036), whereas FEV1 did not change significantly. At 30 days after surgery, marked reductions in FVC and FEV1 persisted compared with the baseline (*p* < 0.001 for both), whereas the Tiffeneau index did not differ significantly from the baseline. No postoperative pulmonary complications were recorded in this cohort; however, postoperative outcomes were descriptive and cannot be attributed to the intervention because no comparator group was included.

**Conclusion:**

In elderly patients awaiting elective cardiac surgery, a 3‐week physiotherapist‐supervised respiratory training protocol was feasible and was associated with improved preoperative FVC. Because of the small sample size, absence of randomization, and lack of a control group, these findings should be interpreted as exploratory and hypothesis‐generating. Larger controlled studies are required to determine whether preoperative respiratory training improves postoperative pulmonary or functional recovery.

## 1 Introduction

Surgical procedures involving a sternotomy and cardiopulmonary bypass profoundly affect thoracic mechanics and lung volumes. This disruption commonly leads to postoperative pulmonary dysfunction, characterized by reduced vital capacity, impaired respiratory muscle function, and the development of atelectasis, all of which are associated with prolonged hospital stays and increased morbidity (Probst et al. 2014; Restrepo et al. 2011; Sweity et al. 2021; Miskovic and Lumb 2017).

To mitigate these risks, preoperative respiratory training (prehabilitation) is increasingly utilized (Restrepo et al. 2011; Sweity et al. 2021). Elderly cardiac surgery patients often present with reduced baseline pulmonary reserve and age‐related decline in respiratory system physiology, making them highly susceptible to surgical trauma (Sharma and Goodwin 2006; Hulzebos et al. 2006). Prehabilitation aims to familiarize patients with breathing exercises and maximize their preoperative lung expansion, theoretically providing a functional buffer against postoperative decline (Restrepo et al. 2011; Hulzebos et al. 2006).

Although respiratory exercises are commonly used in clinical practice, few real‐world studies describe individual longitudinal spirometric trajectories from baseline to post‐prehabilitation and through to the 30‐day postoperative follow‐up in elderly cardiac surgery cohorts. Granular data tracking these mechanical changes over time remain sparse. Therefore, the contribution of the present study is not to establish efficacy, which requires controlled trials, but to describe the feasibility, adherence, and longitudinal spirometric trajectory from baseline to post‐training and 30 days after surgery in a real‐world elderly cardiac surgery cohort.

The primary objective of this study was to evaluate the feasibility and short‐term spirometric response to a 3‐week physiotherapist‐supervised incentive spirometry program in elderly patients awaiting elective cardiac surgery. The secondary objective was to explore demographic and biological factors associated with these pulmonary‐function trajectories.

## 2 Methods

### 2.1 Study Design and Participants

This prospective, single‐center observational study was conducted at the cardiovascular surgery department from March to December 2021. No randomization, control group, or blinding was applied. Therefore, the study was designed as an exploratory feasibility and longitudinal pulmonary‐function trajectory study rather than as a definitive efficacy trial. The study followed the STROBE (Strengthening the Reporting of Observational Studies in Epidemiology) recommendations, was registered at ClinicalTrials.gov (NCT06153550), and was approved by the regional ethics committee (IRBN522021/CHUSTE).

**FIGURE 1:**
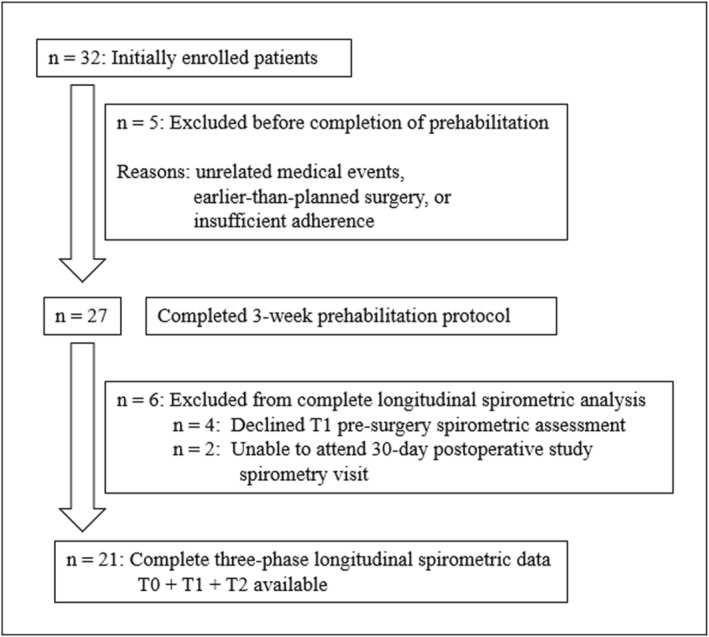
Participant flow diagram. T0, baseline pre‐program spirometric assessment; T1, pre‐surgery spirometric assessment after completion of the 3‐week respiratory training program; T2, 30‐day postoperative study spirometry visit. Among 32 initially enrolled patients, 5 were excluded before completion of the prehabilitation protocol. Twenty‐seven patients completed the prehabilitation protocol. Six patients were excluded from the complete longitudinal spirometric analysis because of incomplete study spirometric assessment: four declined the T1 pre‐surgery spirometric assessment, and two were unable to attend the 30‐day postoperative study spirometry visit. These missing study assessments affected only inclusion in the complete longitudinal spirometric analysis and did not affect planned surgical care or routine clinical management. Therefore, 21 patients had complete T0, T1, and T2 spirometric data available for final analysis.

Patients aged ≥ 65 years scheduled for elective cardiac surgery within 3 weeks were consecutively approached for inclusion. Exclusion criteria included emergency surgery, severe pre‐existing obstructive pulmonary disease, and neuromuscular disorders. Additionally, patients with a Body Mass Index (BMI) > 30 kg/m^2^ were excluded because obesity may independently affect spirometric volumes and chest‐wall mechanics, potentially confounding the spirometric assessments. Out of 32 initially enrolled patients, 5 were excluded before completion of the prehabilitation protocol because of unrelated medical events, earlier‐than‐planned surgery, or insufficient adherence. Ultimately, 27 patients completed the prehabilitation protocol. Among these, 21 patients provided complete three‐phase longitudinal spirometric data for final statistical analysis. Six patients were excluded from the complete longitudinal spirometric analysis due to incomplete study spirometric assessment: Four patients declined the T1 pre‐surgery spirometric assessment, and two patients were unable to attend the 30‐day postoperative study spirometry visit (Figure 1). These missing study assessments affected only inclusion in the complete longitudinal spirometric analysis and did not affect planned surgical care or routine clinical management.

### 2.2 Intervention

Patients underwent a 3‐week respiratory prehabilitation program using the TriFlo 2 (Hudson RCI, Teleflex Medical, USA) incentive spirometer in a home‐based setting prior to admission (Kumar et al. 2016). The protocol required six daily sessions, each comprising 10 repetitions of slow, deep inspirations (Restrepo et al. 2011). The 3‐week duration was selected because it corresponded to the usual preoperative waiting period for elective cardiac surgery in our institution. The frequency of six daily sessions was based on local physiotherapy practice and the clinical objective of repeated low‐intensity inspiratory‐volume practice while maintaining feasibility for elderly patients at home. This dosage should therefore be considered pragmatic and institution‐specific rather than a formally validated dose‐response protocol.

Physiotherapists provided comprehensive, standardized instructions during the initial hospital consultation. To monitor adherence and correct technique, patients were followed twice weekly by physiotherapists via phone or outpatient visits. Most patients were followed through outpatient physiotherapist visits, whereas three patients were monitored remotely by phone because direct outpatient supervision was not feasible. The same standardized written instructions, daily logbooks, and twice‐weekly verification schedule were used for all patients. Compliance was measured using daily patient logs and verified during twice‐weekly physiotherapist check‐ins. Standard postoperative physiotherapy protocols were uniformly applied to all patients following surgery (Probst et al. 2014).

### 2.3 Outcome Measures

Pulmonary function was assessed using a portable AirMed machine (Pond Healthcare Innovation, Sweden). To ensure accuracy, the best of three spirometry attempts was retained when the quality category was A, B, or C, according to ATS/ERS standardization recommendations (Culver et al. 2017). The same device was used for all measurements. Spirometric volumes were expressed in liters.

Measurements were recorded at three specific time points:

1. **T0:** Baseline (Pre‐program)
2. **T1:** Pre‐surgery (performed during hospital admission after completion of the 3‐week training program and before surgery)
3. **T2:** Post‐surgery (30‐day follow‐up)

Primary outcome parameters were forced vital capacity (FVC), forced expiratory volume in 1 s (FEV1), and the Tiffeneau index (calculated as FEV1/FVC × 100). The Tiffeneau index was reported as an absolute ratio expressed as a percentage and not as percentage‐predicted spirometry.

Secondary clinical outcomes were collected to contextualize the postoperative recovery trajectory. Intraoperative variables recorded included total cardiopulmonary bypass (CPB) time (minutes) and aortic cross‐clamp time (minutes). Postoperative recovery metrics included Intensive Care Unit (ICU) length of stay (days) and total hospital length of stay (days). Postoperative pulmonary complications (PPCs) were defined as pneumonia, atelectasis, or radiographic lung consolidation. PPCs were assessed during postoperative follow‐up using clinical evaluation and routine postoperative chest X‐rays.

### 2.4 Statistical Analysis

Data distribution was assessed for normality. Exploratory association analyses were performed using Pearson or Spearman correlation coefficients, depending on the data distribution, to evaluate the relationships between spirometric responses and demographic/biological variables. No multivariable regression model was retained in the revised analysis because of the limited sample size and the risk of unstable estimates. Paired comparisons between time points (T0 vs. T1 and T0 vs. T2) were analyzed using paired Student's *t*‐tests. When normality was uncertain, non‐parametric analyses were considered as sensitivity analyses. No formal sample‐size calculation was performed because this was an exploratory feasibility study based on consecutively available patients during the study period. Therefore, all statistical comparisons and association analyses should be interpreted as exploratory and hypothesis‐generating. A *p*‐value < 0.05 was considered statistically significant.

## 3 Results

### 3.1 Participant Characteristics and Compliance

The 3‐week prehabilitation protocol was feasible. Among the 27 patients who completed the pre‐surgical phase, adherence was high, with patients completing ≥ 80% of their expected daily sessions based on logbook verification. No adverse events related to incentive‐spirometry exercises were reported. Patients reported a mean subjective satisfaction score of 8/10 regarding the protocol. The cohort presented substantial clinical heterogeneity and cardiac burden: 13 patients (48%) had coronary artery disease and 24 patients (88%) had valvular heart disease, including 17 patients with aortic valve disease and 7 with mitral valve disease. The mean ejection fraction was 59.2 ± 7.9%, and the mean pulmonary artery pressure was 34.8 ± 12.7 mmHg (Table 1). The completed cohort had a mean age of 71.8 ± 6.7 years, and 20 patients (74%) were male.

**TABLE 1:** Clinical and demographic characteristics (*n* = 27).

Characteristic	Value
Age, years, mean ± SD	71.8 ± 6.7
Male sex, *n* (%)	20/27 (74%)
BMI, kg/m^2^, mean ± SD	25.1 ± 5.1
Current smoking, *n* (%)	0/27 (0%)
Former smoking, *n* (%)	7/27 (26%)
Hypertension, *n* (%)	18/27 (67%)
Coronary artery disease, *n* (%)	13/27 (48%)
Valvular heart disease, *n* (%)	24/27 (88%)
Aortic valve disease among patients with valvular heart disease, *n* (%)	17/24 (71%)
Mitral valve disease among patients with valvular heart disease, *n* (%)	7/24 (29%)
Ejection fraction, %, mean ± SD	59.2 ± 7.9
Pulmonary artery pressure, mmHg, mean ± SD	34.8 ± 12.7
Beta‐blocker therapy, *n* (%)	23/27 (85%)
ACE inhibitor therapy, *n* (%)	20/27 (74%)

Abbreviations: ACE, angiotensin‐converting enzyme; BMI, body mass index; SD, standard deviation.

### 3.2 Longitudinal Pulmonary Function

FVC increased significantly after the 3‐week respiratory training program (4.22 ± 1.16 L to 5.09 ± 1.96 L; *p* = 0.036), whereas FEV1 did not change significantly. At 30 days after surgery, both FVC and FEV1 were significantly lower than baseline, reflecting the expected physiological impact of sternotomy. The Tiffeneau index did not differ significantly between the baseline and 30 days (Table 2).

**TABLE 2:** Evolution of pulmonary function across the three study phases (*n* = 21).

Parameter	T0	T1	Δ T1–T0	95% CI	dz	*p*	T2	Δ T2–T0	95% CI	dz	*p*
FVC (L)	4.22 ± 1.16	5.09 ± 1.96	+0.87	0.07 to 1.68	0.49	0.036	2.57 ± 1.03	−1.65	−2.01 to −1.30	−2.12	< 0.001
FEV1 (L)	2.94 ± 0.82	3.06 ± 0.98	+0.12	−0.18 to 0.42	0.18	0.409	1.80 ± 0.69	−1.14	−1.51 to −0.77	−1.41	< 0.001
Tiffeneau index (%)	70.41 ± 10.13	64.45 ± 17.02	−5.96	−13.39 to 1.46	−0.37	0.109	71.82 ± 9.88	+1.41	−4.48 to 7.29	0.11	0.624

*Note:* Values are mean ± SD. Δ indicates mean paired change. *p*‐values are from paired *t*‐tests.

Abbreviations: CI, confidence interval; dz, Cohen's dz effect size.

### 3.3 Surgical and Postoperative Clinical Outcomes

The cohort underwent cardiac surgery with substantial intraoperative burden, with a mean aortic cross‐clamp time of 80.84 ± 35.87 min and a mean cardiopulmonary bypass time of 104.88 ± 47.29 min. Postoperative outcomes are reported descriptively because the study did not include a comparator group. No postoperative pulmonary complications were recorded during follow‐up, and no pneumonia, atelectasis, or radiographic lung consolidation was identified on routine postoperative chest X‐rays. Mean ICU stay was 2.17 ± 0.7 days, and mean total hospital stay was 6.71 ± 2.35 days.

### 3.4 Exploratory Associations With Spirometric Response

Exploratory analyses of demographic variables revealed that advanced age was negatively correlated with baseline FEV1 and the Tiffeneau index. Additionally, males exhibited higher absolute baseline and postoperative FEV1 values than females. These demographic correlations reflect the expected physiological baseline differences in absolute lung volumes rather than differences in the response to prehabilitation. Because of the small number of female participants, these sex‐related findings should be considered descriptive only. No conclusion regarding sex‐specific response to respiratory training can be drawn from this small and imbalanced cohort. Increased BMI showed a non‐significant negative trend with the postoperative Tiffeneau index (Table 3). Furthermore, exploratory analysis indicated that baseline hematocrit was positively associated with FEV1 improvement during the training phase (*r* = 0.552, *p* = 0.003).

**TABLE 3:** Impact of demographic variables on pulmonary trajectories.

Demographic factor	Predicted metric	Correlation/Trend	*p*‐value
Age	Baseline FEV1	*r* = −0.525 (negative)	0.003
Age	Baseline Tiffeneau index	*r* = −0.436 (negative)	0.016
Sex	Baseline FEV1	Higher in males	0.009
Sex	Postoperative FEV1 (30 days)	Higher in males	0.030
BMI	Postoperative Tiffeneau index	*r* = −0.357 (negative)	0.094

## 4 Discussion

This prospective observational study found that a 3‐week physiotherapist‐supervised incentive‐spirometry protocol was feasible in elderly patients awaiting elective cardiac surgery and was associated with a significant preoperative increase in FVC. Marked reductions in FVC and FEV1 were observed 30 days after surgery, consistent with the expected postoperative impact of cardiac surgery. No postoperative pulmonary complications were recorded in this small selected cohort; these postoperative observations remain descriptive in the absence of a comparator group (Probst et al. 2014; Miskovic and Lumb 2017; Hulzebos et al. 2006).

The increase in FVC after training may reflect improved inspiratory effort, thoracic expansion, respiratory muscle coordination, better familiarity with spirometric maneuvers, or mobilization of pulmonary volumes (Restrepo et al. 2011; Kumar et al. 2016). The use of standardized repeated spirometry with the same device supports the internal consistency of the observed longitudinal changes. Because this study did not directly measure respiratory muscle strength, lung compliance, gas exchange, or imaging‐based lung recruitment, the observed FVC improvement should not be interpreted as direct proof of alveolar recruitment or postoperative protection (Sweity et al. 2021; Hulzebos et al. 2006).

The surgical burden was substantial, as reflected by the mean aortic cross‐clamp time of 80.84 ± 35.87 min and mean cardiopulmonary bypass time of 104.88 ± 47.29 min. The postoperative reductions in FVC and FEV1 are therefore consistent with the known inflammatory and mechanical effects of cardiac surgery, including sternotomy‐related chest‐wall impairment, anesthesia‐related atelectasis, postoperative pain, and cardiopulmonary bypass‐associated inflammatory responses (Probst et al. 2014; Miskovic and Lumb 2017).

The association between baseline hematocrit and FEV1 response during training is hypothesis‐generating. It may reflect oxygen‐carrying capacity, underlying physiological reserve, or comorbidity burden rather than a direct causal effect. This finding requires confirmation in larger controlled studies before clinical applications can be recommended.

Overall, these findings support the feasibility of systematic respiratory assessment before and after cardiac surgery. Future controlled studies should determine whether preoperative spirometric changes translate into clinically meaningful outcomes, including postoperative pulmonary complications, exercise capacity, dyspnea, quality of life, and patient‐reported recovery.

### 4.1 Limitations

This study has several limitations. First, the absence of a control group, randomization, and blinding limits causal interpretation and may have introduced selection, performance, and measurement bias. Second, the sample size was small, with only 21 patients providing complete three‐phase longitudinal spirometric data, and no formal sample‐size calculation was performed. This limited the power for subgroup, multivariable, and adjusted analyses. Third, the cohort was clinically heterogeneous, including patients with coronary artery disease and valvular heart disease involving aortic and mitral valve disease. These conditions may represent different baseline cardiopulmonary risk profiles, surgical procedures, and perioperative recovery trajectories. Because of the limited sample size, we could not reliably adjust for cardiac diagnosis, surgical complexity, or baseline cardiopulmonary reserve. Fourth, baseline functional capacity, frailty, dyspnea severity, respiratory muscle strength, detailed pulmonary reserve, and quality of life were not assessed. These factors may have influenced both spirometric changes and postoperative recovery. Fifth, adherence was assessed using patient self‐reported logbooks and verified during twice‐weekly physiotherapist follow‐up. Although practical and clinically feasible, self‐reported adherence may be subject to social desirability and overestimation bias. In addition, the use of both outpatient and remote phone follow‐up may have introduced variability in supervision intensity and adherence verification. Sixth, the cohort was predominantly male, with only seven female participants, preventing reliable interpretation of sex‐related differences. Finally, external validity is limited because patients with BMI > 30 kg/m^2^, severe pre‐existing obstructive pulmonary disease, neuromuscular disorders, emergency surgery, or more severe clinical impairment were excluded.

### 4.2 Implications for Physiotherapy Practice

This exploratory study suggests that a 3‐week physiotherapist‐supervised respiratory training program using incentive spirometry is feasible in selected elderly patients awaiting elective cardiac surgery. The protocol may help structure preoperative patient education, familiarization with breathing exercises, and adherence monitoring. These findings should be considered preliminary and should not be interpreted as evidence that the intervention reduces postoperative pulmonary complications or improves recovery. Further controlled studies are required before broad clinical recommendations can be made.

## 5 Conclusion

In elderly patients awaiting elective cardiac surgery, a 3‐week physiotherapist‐supervised respiratory training protocol was feasible and was associated with improved preoperative FVC. No postoperative pulmonary complications were recorded in this selected cohort, but postoperative outcomes remain descriptive and cannot be attributed to the intervention. Larger controlled studies are required to determine whether preoperative respiratory training reduces postoperative pulmonary complications or improves functional recovery and patient‐reported outcomes.

## Author Contributions

**Andranik Petrosyan:** conceptualization, methodology, data curation, writing – original draft preparation, visualization, investigation. **Emilie Besson:** conceptualization, methodology, data curation, visualization, investigation. **Nathalie Grand:** software, supervision. **Julien Lanoiselee:** methodology, software. **Khalil Raissouni:** visualization, investigation. **Kasra Azarnoush:** validation, writing – reviewing and editing.

## Funding

The authors have nothing to report.

## Ethics Statement

The study was approved by the regional ethics committee of the university hospital with the following approval number—*IRBN522021/CHUSTE,* and is registered in ClinicalTrials.gov with ID number NCT06153550.

## Consent

Before participating in this study, patients were provided with written information and gave their informed consent.

## Conflicts of Interest

The authors declare no conflicts of interest.

## Data Availability

The data that support the findings of this study are available on request from the corresponding author. The data are not publicly available due to privacy or ethical restrictions.
